# The genetic architecture of amino acids dissection by association and linkage analysis in maize

**DOI:** 10.1111/pbi.12712

**Published:** 2017-04-05

**Authors:** Min Deng, Dongqin Li, Jingyun Luo, Yingjie Xiao, Haijun Liu, Qingchun Pan, Xuehai Zhang, Minliang Jin, Mingchao Zhao, Jianbing Yan

**Affiliations:** ^1^ National Key Laboratory of Crop Genetic Improvement Huazhong Agricultural University Wuhan China

**Keywords:** amino acid, Quality Protein Maize (QPM), co‐expression network, GWAS, linkage mapping, metabolism

## Abstract

Amino acids are both constituents of proteins, providing the essential nutrition for humans and animals, and signalling molecules regulating the growth and development of plants. Most cultivars of maize are deficient in essential amino acids such as lysine and tryptophan. Here, we measured the levels of 17 different total amino acids, and created 48 derived traits in mature kernels from a maize diversity inbred collection and three recombinant inbred line (RIL) populations. By GWAS, 247 and 281 significant loci were identified in two different environments, 5.1 and 4.4 loci for each trait, explaining 7.44% and 7.90% phenotypic variation for each locus in average, respectively. By linkage mapping, 89, 150 and 165 QTLs were identified in B73/By804, Kui3/B77 and Zong3/Yu87‐1 RIL populations, 2.0, 2.7 and 2.8 QTLs for each trait, explaining 13.6%, 16.4% and 21.4% phenotypic variation for each QTL in average, respectively. It implies that the genetic architecture of amino acids is relative simple and controlled by limited loci. About 43.2% of the loci identified by GWAS were verified by expression QTL, and 17 loci overlapped with mapped QTLs in the three RIL populations. GRMZM2G015534, GRMZM2G143008 and one QTL were further validated using molecular approaches. The amino acid biosynthetic and catabolic pathways were reconstructed on the basis of candidate genes proposed in this study. Our results provide insights into the genetic basis of amino acid biosynthesis in maize kernels and may facilitate marker‐based breeding for quality protein maize.

## Introduction

Maize (*Zea mays*) is one of the most widely grown crops worldwide. It is not only a staple food for people and animals, but also an important industrial material for fuel and other applications. Typically, the maize endosperm is ~10% protein, and seed storage proteins supply nitrogen for the germinating seedling and are also an important protein source for humans and animals. The amino acid composition and quantity of seed storage proteins are related to the nutritional quality of seeds (Mandal and Mandal, 2000; Young and Pellett, 1994). However, the maize cultivars widely planted usually have insufficient levels of essential amino acids, such as lysine and tryptophan (Misra *et al*., 1972). In order to facilitate breeding for balanced amino acid composition, it is important to identify the genes controlling amino acid content in the maize kernel.

Although more than 180 amino acids have been discovered in nature, only 20 amino acids constitute proteins. Many amino acids, such as homoserine, homocysteine, ornithine and citrulline, play important roles in growth and development (Dunlop *et al*., 2015), defence against insect herbivores (Huang *et al*., 2011). Amino acids are also important signalling molecules regulating several signal pathways related to the growth and development of both animals and plants. Some studies have found that aspartate plays an important role in human cell proliferation (Birsoy *et al*., 2015; Sullivan *et al*., 2015). Proline could maintain cellular osmotic homoeostasis, as well as redox balance and energy status (Krishnan *et al*., 2008). Proline also may function as a molecular chaperone to protect proteins from denaturation (Mishra and Dubey, 2006; Sharma and Dubey, 2005), an antioxidant to scavenge ROS, a singlet oxygen quencher (Matysik *et al*., 2002; Smirnoff and Cumbes, 1989), or a regulator of the cell cycle in maize (Wang *et al*., 2014).

The amino acid metabolism pathways, including biosynthesis, degradation and regulation, are well studied in microorganisms (Miflin and Lea, 1977; Umbarger, 1969, 1978). Studies of the model plant *Arabidopsis thaliana* have focused on the roles of amino acids in nitrogen nutrition (Crawford and Forde, 2002), N‐assimilation (Coruzzi, 2003), metabolism and regulation (Hell and Wirtz, 2011; Ingle, 2011; Jander and Joshi, 2009; Tzin and Galili, 2010a; Verslues and Sharma, 2010). Some key genes regulating free amino acid content have been identified in *Arabidopsis* (Angelovici *et al*., 2013), tobacco (Maloney *et al*., 2010), soya bean (Ishimoto *et al*., 2010; Takahashi *et al*., 2003), rapeseed (Moulin *et al*., 2000, 2006), rice (Kang *et al*., 2005; Zhou *et al*., 2009) and maize (Mertz *et al*., 1964; Muehlbauer *et al*., 1994; Shaver *et al*., 1996; Wang *et al*., 2001, 2007). Opaque2 (O2) is an endosperm‐specific transcription factor belonging to the bZIP family, whose mutation could increase free lysine levels and enhance the overall nutritional value of grain by reducing the 22‐kD α‐ and β‐zein transcripts and proteins in maize (Hunter *et al*., 2002; Kodrzycki *et al*., 1989; Mertz *et al*., 1964). Due to the lysine content in *o2* mutant maize kernels being 70% higher than wild type, it has become a subject of intense research over the past several decades (Wu and Messing, 2014). However, the *o2* gene has not been widely used for breeding high‐nutrition maize lines because its pleiotropic effects are negatively associated with agronomic performance (Loesch *et al*., 1976; Nass and Crane, 1970; Zhang *et al*., 2016). Identification of more favourable genes and increasing the understanding of the underlying amino acid biosynthetic pathways are the key steps for breeding maize with high‐quality protein (Ufaz and Galili, 2008).

With the rapid development of DNA and RNA‐sequencing technologies, high‐density genotyping with SNPs became easily accessible, enabling genomewide association studies (GWAS). This method became a powerful tool for complex trait dissection in plants (Xiao *et al*., 2016; Yan *et al*., 2011). Many GWAS were performed in plants including maize (Li *et al*., 2013; Xiao *et al*., 2016), rice (Huang *et al*., 2010, 2012), canola (Liu *et al*., 2016d; Luo *et al*., 2015), sorghum (Morris *et al*., 2013), foxtail millet (Jia *et al*., 2013), *Arabidopsis* (Atwell *et al*., 2010) and others. Recently, the expression data of 28 769 genes and 1.03 million high‐quality SNPs were obtained by deep RNA‐sequencing of the immature seeds at 15 days after pollination of 368 diverse maize inbred lines (Fu *et al*., 2013). These data were used for studies of maize quality traits, including oil concentration (Li *et al*., 2013), vitamin E content (Li *et al*., 2012b) and metabolites (Wen *et al*., 2014). They provide a valuable resource for studying the genetic architecture of maize quantitative traits.

To better understand the genetic components underlying the natural variation and the metabolism of amino acids in the maize kernel, we used an automatic amino acid analyser to quantify the total amino acids of mature maize kernel from a diversity association panel of 513 lines (Yang *et al*., 2011, 2014) and three RIL populations (Pan *et al*., 2016). GWAS and linkage mapping were combined to dissect the genetic architecture of amino acids in the maize kernel. Many previously known and unknown genes directly or indirectly involved in amino acid metabolism were identified, which has helped to ascertain the amino acid metabolism network. Some of the candidate genes were validated by multiple approaches, including expression QTL mapping, QTL fine mapping, bioinformatics, and further confirmed by genetic transformation. These results provide new insights for understanding amino acid biosynthesis and thus enhancing the breeding of high‐nutrition maize.

## Results

### Natural variation of amino acids in maize kernel

Using an automatic amino acid analyzer L‐8800 (L‐8800, Hitachi Instruments Engineering, Tokyo, Japan), we assessed the variation in total amino acid content in dry matured maize kernels, which included an association panel (513 inbred lines) harvested from two environments and three RIL populations (169, 152, 146 lines for B73/BY804 (BB), KUI3/B77 (KB) and ZONG3/YU87‐1 (ZY), respectively). The concentrations of seventeen amino acids (Ala, Arg, Asx, Glx, Gly, Lle, Leu, Lys, Met, Pro, Phe, Val, Tyr, His, Cys, Thr and Ser in mg/g dry maize kernel) and total amino acid concentration (sum of the seventeen amino acids) were calculated. Forty‐seven derived compositional traits were also calculated (detailed in methods). The level of each amino acid‐related trait varied widely in both the association panel and three RIL populations (Figure S1). Variation ranged from a 1.2‐fold difference in Phe/PT to 14.9‐fold difference in Cys/Total, and 1.1‐fold difference in GT/Total and Glx/GT to 5.7‐fold difference in Met/Total in association and linkage mapping populations, respectively (Tables S1, S2). For the average total lysine content, the maximum ratio of 3.1‐fold difference was found in the KB population (1.72–5.37 mg/g). The skewness, kurtosis and other detailed information for each amino acid are shown in Tables S1 and S2.

### Loci associated with amino acid content identified by GWAS and linkage mapping

GWAS was performed using an association panel including 513 maize diverse inbred lines (Yang *et al*., 2011, 2014) and 1.25 million high‐quality single nucleotide polymorphisms (SNPs) with minor allele frequency (MAF) >0.05 (Fu *et al*., 2013; Liu *et al*., 2016a). In total, 247 and 281 associated loci were identified in AM1 and AM2 at *P* ≤ 2.04 × 10^−6^, with an average of 5.1 and 4.4 loci for each trait, respectively (Table 1, Figure S2, Table S3). The phenotypic variation explained by each locus for each amino acid trait ranged from 5.21% (Ala/AT in AM2) to 19.74% (Leu/Total in AM1), with an average of 7.44% for AM1 and 7.90% for AM2 (Figure S3, Table S3). Ten loci with effects greater than 15% were identified in two environments. For each trait, the total phenotypic variation explained by all the identified loci was 23.3% (ranged from 5.6% to 66.3%) and 19.3% (ranged from 5.4% to 49.5%) in AM1 and AM2, respectively.

**Table 1:** Summary of significant loci–trait associations identified by GWAS and QTL by linkage mapping

Population^a^	BB	KB	ZY	AM1	AM2
Number of Traits with QTL^b^	45	56	59	48	64
Number of Loci^c^	89	150	165	247	281
Average loci per trait^d^	2.0 ± 1.2	2.7 ± 1.5	2.8 ± 1.6	5.1 ± 6.9	4.4 ± 3.3

a BB, KB, ZY represent three linkage populations B73/By804, Kui3/B77, Zong3/Yu87‐1, respectively; AM1, AM2 represent the two environments.

b Number of traits with QTLs identified. 65 amino acids traits were analysed in each population.

c Number of significant loci detected on the association panel (*P *≤* *2.04 × 10^−6^, MLM) and a uniform threshold for significant QTLs was determined by 500 permutations (*P* = 0.05).

d Average number of significant loci (or QTL) detected per trait ± S.D.

Three RIL populations (BB, KB and ZY) were genotyped with high‐density SNP array (Pan *et al*., 2016) and were used for QTL mapping for the amino acid traits. At least one QTL was identified for 45, 56, 59 among 65 measured traits in BB, KB and ZY RIL populations, respectively. In total, 89, 150, and 165 QTLs were identified for BB, KB, and ZY populations with an average of 2.0, 2.7 and 2.8 QTLs for each trait, respectively (Table 1, Figure S2, Table S4). For the same trait, only 15 QTLs were detected in more than one population, implying that different low‐frequency QTL existed in different genetic backgrounds (Xiao *et al*., 2016). Each QTL explained the phenotypic variation of 6.40%–14.88% (BB), 3.42%–16.96% (KB), and 5.87%–23.32% (ZY), with an average of 9.03%, 9.39% and 10.15%, respectively (Figure S3, Table S4). Thirteen QTLs with effects greater than 15% were identified in the three RIL populations. For each trait, all the identified QTLs on average explained 13.6% (ranged from 7.2% to 32.6%), 16.4% (ranged from 4.9% to 32.4%) and 21.4% (ranged from 8.5% to 49.9%) of the total phenotypic variance in BB, KB and ZY RIL population, respectively.

### Candidate genes and QTL hotspots

**Figure 1:**
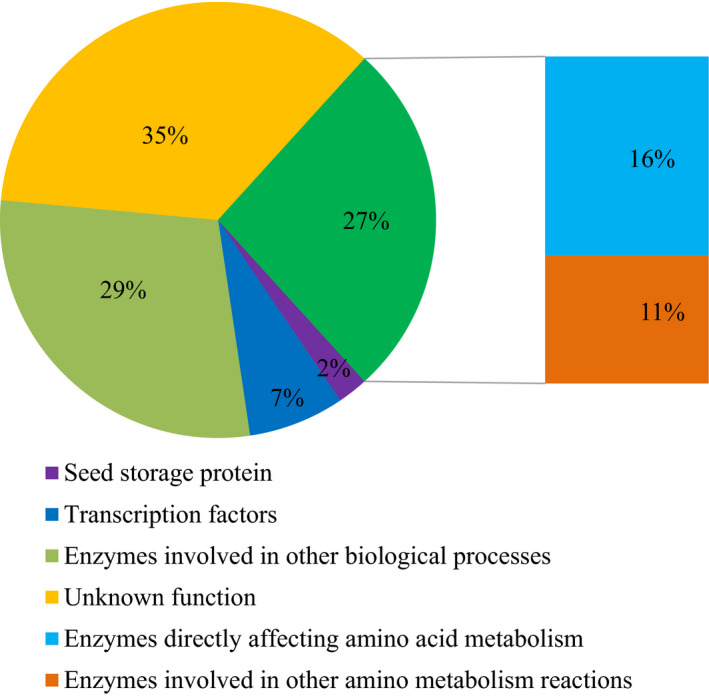
Functional category annotations for 308 candidate genes and their respective percentages identified via GWAS as significantly associated with amino acid traits in maize kernels.

Subsequently, limited overlaps were found between the loci (17/528) identified by GWAS and the QTLs identified by linkage mapping for the same trait in the present study. A total of 308 unique candidate genes corresponding to 528 trait–locus associations identified in two experiments were annotated, and other potential candidate genes within 200 kb (100 kb upstream and downstream of the lead SNPs) of the 528 loci were also listed in Table S3. Among the candidate genes, those encoding enzymes or other protein directly or indirectly affecting amino acid metabolism accounted for 27%, the enzymes involved in other biological processes accounted for 29%, and the functions were unknown for 35%, based on the current database (Figure 1). Gene Ontology (GO) term analysis revealed significant enrichment in terms relating to cellular nitrogen metabolism, amine metabolism, amino acid and derivative metabolism, organic acids and other processes (Figure S4). Expression QTLs (eQTL, *n *= 368) were identified for a plurality of these candidate genes (43.2%, or 133/308) using the previous RNA‐sequencing data of immature kernels (Fu *et al*., 2013). Significant correlations (*P *< 0.05, *n* = 295–326) between the expression level of the candidate genes with eQTLs identified and the phenotypic variation of the corresponding amino acid traits were found in 50 cases (16.2%) (Table S3), which suggests that some of these loci affect phenotypic variation via transcriptional regulation.

**Figure 2:**
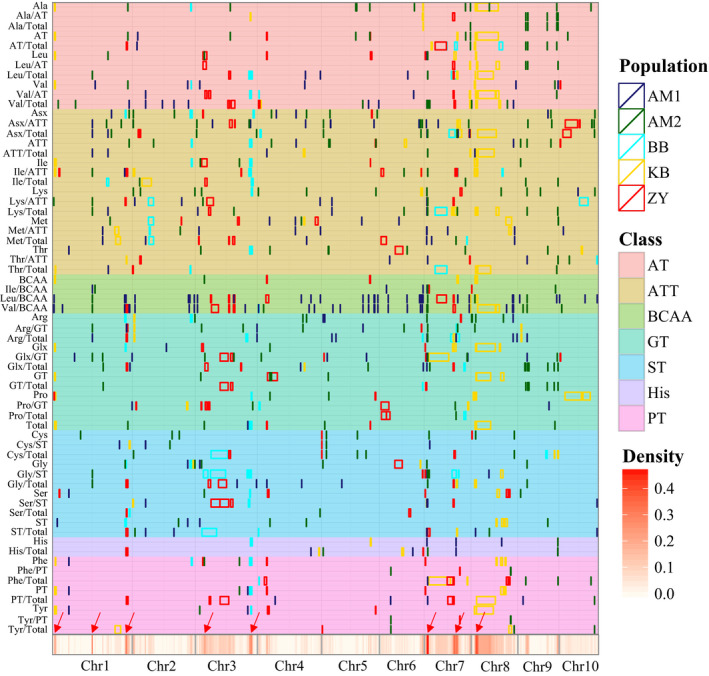
Chromosomal distribution of amino acids loci and QTLs identified in this study. QTL regions (represented by the confidence interval for linkage mapping and the 100 kb up‐ and downstream of the lead SNP for association mapping) across the maize genome responsible for amino acid levels from the different populations are shown as midnight blue (BB), green (AM1), cyan (AM2) gold (KB) and red (ZY) boxes, respectively. The class represents different amino acid families. AT, pyruvate‐derived amino acid family related traits; ATT, aspartate‐derived amino acid family related traits; BCAA, branched‐chain amino acid family related traits; GT, glutamate‐derived amino acid family related traits; PT, phenylalanine‐derived amino acid family related traits; ST, serine‐derived amino acid family related traits; His, histidine family related traits. The *x*‐axis indicates the genetic positions across the maize genome in Mb. Heatmap under the *x*‐axis illustrates the density of amino acid loci and QTLs across the genome. The red arrows show the QTL hotspots. The detailed information of all detected loci and QTLs is shown in Tables S3 and S4. Amino acid traits from different derived families are marked by distinct colours as shown on the right.

QTLs were not distributed evenly on the chromosomes, based on 1000‐time permutation tests at the level of 0.05, and eight QTL hotspots were observed on chromosomes 1, 3, 7, 8 (Figure 2, Tables S3, S4). These QTLs were often shared by biologically related amino acids. For example, the QTLs affecting Leu, Val, and Ile contents or derived traits were enriched on chromosome 7. The candidate genes underlying these QTL hotspots could include regulators of the metabolic pathway, and influence the rate‐limiting reactions. Interestingly, two QTL hotspots (on chromosome 3 and 7) overlapped with the metabolite QTL hotspot identified in a previous study using three different tissues from the BB population in (Wen *et al*., 2015), which helps identify the underlying genes and their regulating pathway.

### Amino acid metabolic network involving identified genes and their co‐expression genes

**Figure 3:**
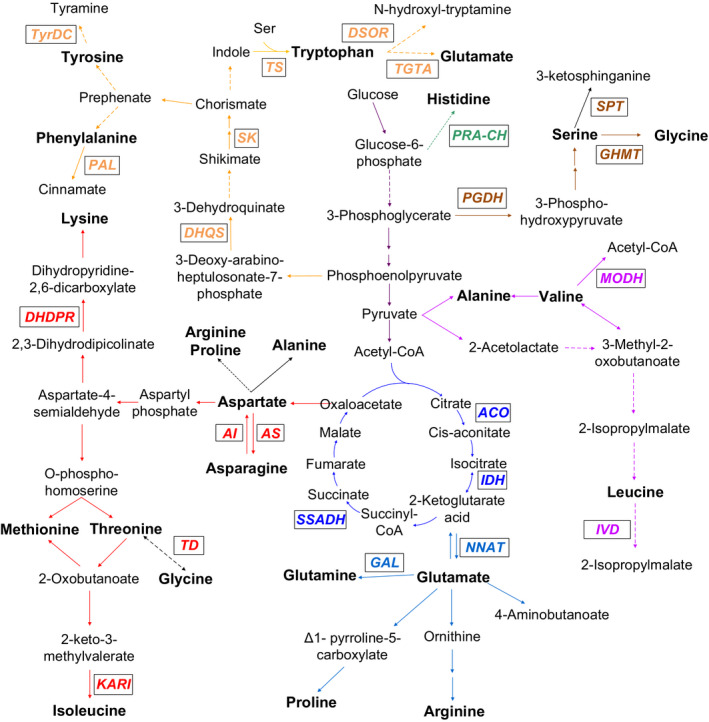
A maize amino acids network involving key genes identified in this study by GWAS. The different colours represent the different amino acids families. The purple, sky‐blue, red, brown, dark green, orange lines represent the metabolism pathway of pyruvate‐derived, glutamate‐derived, aspartate‐derived, serine‐derived, Histidine, phenylalanine‐derived amino acids, respectively. The blue lines represent the TCA cycle. Candidate genes identified in this study by GWAS are shown in the respective pathway. KARI, Ketol‐acid reductoisomerase; GHMT, Glycine hydroxymethyltransferase; SPT, Serine palmitoyltransferase; PGDH, Phosphoglycerate dehydrogenase; IDH, Isocitrate dehydrogenase; TS, Tryptophan synthase; TGTA, Tryptophan Glutamate transaminase; DSOR, Disulphide oxidoreductase; MODH, 3‐methyl‐2‐oxobutanoate dehydrogenase; IVD, Isovaleryl‐CoA dehydrogenase; DHDPR, Dihydrodipicolinate reductase; TD, L‐threonine 3‐dehydrogenase; AS, Asparagine synthase; AI, Asparaginase; SK, Shikimate kinase; PAL, Phenylalanine ammonia‐lyase; GAL, Glutamate‐ammonia ligase; NNAT, Nicotianamine aminotransferase; ACO, Aconitate hydratase; SSADH, Succinate semialdehyde dehydrogenase; DHQS, 3‐dehydroquinate synthase; TyrDC, Tyrosine decarboxylase; PRA‐CH, Phosphoribosyl‐AMP cyclohydrolase.

We reconstructed a maize amino acid metabolism network based on the published results in *Arabidopsis* (Coruzzi, 2003; Hell and Wirtz, 2011; Ingle, 2011; Jander and Joshi, 2009; Tzin and Galili, 2010a,b; Verslues and Sharma, 2010) and data obtained from this study. Notably, 23 candidate genes involved in amino acid anabolism and catabolism were identified by GWAS (Figure 3, Table 2). Five of 23 genes have been reported previously in maize, including isocitrate dehydrogenase (*IDH*) (Curry and Ting, 1976; Zhang *et al*., 2010), phenylalanine ammonia‐lyase (*PAL*) (Havir, 1971) tryptophan synthase (*TS*) (Wright *et al*., 1992), asparagine synthase (*AS*) (Chevalier *et al*., 1996; Schmidt *et al*., 1987) and aconitate hydratase (*ACO*) (Wendel *et al*., 1988). The remaining candidate genes identified in this study may be involved in amino acid biosynthetic pathways, based on the available database annotation and comparative genomic approaches although the functions have not been fully explored in maize (Table 2).

**Table 2:** SNPs and candidate genes significantly associated with amino acid traits and were used in the amino acids network analysis

Candidate Gene^a^	Lead SNP	Chromosome	Position (bp)^b^	Allele	MAF^c^	*P* value^d^	*R* ^2^ (%)^e^	*P* value (eQTL)^f^	Correlation (Phenotype vs expression)^g^	Annotation
GRMZM2G373859	chr1.S_210471253	1	210471253	T/C	0.241	5.35E‐08	9.59	NS		Glutamine dumper
GRMZM2G081886	chr2.S_5014818	2	5014818	C/A	0.169	1.75E‐06	5.15	2.17E‐11	−0.206	Phosphoglycerate dehydrogenase
GRMZM2G139463	chr2.S_20803501	2	20803501	C/T	0.201	1.34E‐06	8.56	6.00E‐34	−0.096	L‐asparaginase
GRMZM2G118345	chr2.S_28051836	2	28051836	G/T	0.395	1.61E‐06	6.70	1.76E‐21	−0.151	Phenylalanine ammonia‐lyase
GRMZM2G161868	chr3.S_202363139	3	202363139	A/C	0.208	7.05E‐07	7.07	NS		Ketol‐acid reductoisomerase
GRMZM2G006480	chr4.S_3888039	4	3888039	G/A	0.412	9.23E‐07	6.95	NS		Nicotianamine aminotransferase
GRMZM2G169593	chr4.S_35917250	4	35917250	C/T	0.085	3.39E‐07	6.78	NS		Tryptophan synthase
GRMZM2G090241	chr4.S_53259978	4	53259978	C/T	0.093	1.71E‐06	5.96	NS		Dihydrodipicolinate reductase
GRMZM2G036464	chr4.S_167077316	4	167077316	C/T	0.084	1.11E‐06	5.98	4.15E‐17	−0.051	Glutamate‐ammonia ligase
GRMZM2G119482	chr4.S_236213884	4	236213884	T/A	0.052	9.56E‐07	11.34	NS		Succinate semialdehyde dehydrogenase
GRMZM2G381051	chr6.S_27117945	6	27117945	A/G	0.109	1.10E‐06	5.74	NS		Isovaleryl‐CoA dehydrogenase
GRMZM2G009400	chr6.S_158268310	6	158268310	C/G	0.083	1.71E‐06	10.84	NS		Tyrosine decarboxylase
GRMZM5G829778	chr6.S_165625349	6	165625349	C/T	0.181	5.34E‐07	5.91	3.23E‐19	−0.114	Isocitrate dehydrogenase (NADP(+))
GRMZM2G015534	chr7.S_10695002	7	10695002	C/G	0.105	2.36E‐07	7.09	1.58E‐08	−0.270	Opaque 2
GRMZM2G138727	chr7.S_120252509	7	120252509	G/T	0.498	3.43E‐09	8.61	1.40E‐13	0.428	Glutelin‐2 Precursor (27 kDa zein)
GRMZM2G082214	chr8.S_2198542	8	2198542	T/C	0.081	1.48E‐06	7.42	1.78E‐12	0.069	Phosphoribosyl‐AMP cyclohydrolase
GRMZM2G127308	chr8.S_16853869	8	16853869	G/A	0.185	1.33E‐06	8.61	NS		Tryptophan transaminase
GRMZM2G010202	chr8.S_159961615	8	159961615	G/A	0.197	5.72E‐07	5.79	1.24E‐20	−0.134	Serine palmitoyltransferase
GRMZM2G004824	chr9.S_31098627	9	31098627	G/A	0.454	1.80E‐06	5.16	NS		Glycine hydroxymethyltransferase
GRMZM2G078472	chr9.S_137749795	9	137749795	T/C	0.051	1.41E‐06	9.32	NS		Asparagine synthase (glutamine‐hydrolysing)
GRMZM2G009808	chr9.S_151665914	9	151665914	G/A	0.361	1.60E‐06	6.36	NS		Aconitate hydratase
GRMZM2G091819	chr10.S_16572309	10	16572309	C/G	0.116	1.86E‐06	5.48	NS		Disulphide oxidoreductase
GRMZM2G147191	chr7.S_126350017	7	126350017	T/C	0.057	6.88E‐07	6.527	NS		L‐threonine 3‐dehydrogenase
GRMZM2G139412	chr5.S_151841984	5	151841984	G/A	0.116	2.51E‐07	12.338	NS		Shikimate kinase
GRMZM2G037614	chr2.S_233675625	2	233675625	G/A	0.059	9.88E‐07	5.6551	5.75E‐10	−0.102	3‐methyl‐2‐oxobutanoate dehydrogenase
GRMZM2G178826	chr9.S_151366977	9	151366977	A/G	0.417	6.06E‐09	9.0918	1.76E‐22	0.060	3‐dehydroquinate synthase

a A plausible biological candidate gene in the locus or the nearest annotated gene to the lead SNP.

b Position in base pairs for the lead SNP according to version 5b.60 of the maize reference sequence.

c Minor allele frequency of the lead SNP.

d *P* value of the corresponding metabolic trait calculated by MLM.

e The phenotypic variance explained by the corresponding locus.

f *P* value of the expression QTL of the candidate gene. The *P* value is the lead SNP of eQTL rather than the GWAS lead SNP. NS, not significant; ND, the expression of the candidate gene is not detected. *P* value was calculated by MLM, the sample size *N* = 368.

g Pearson correlation between the expression amount and the phenotypic data of the corresponding metabolic trait.

**Figure 4:**
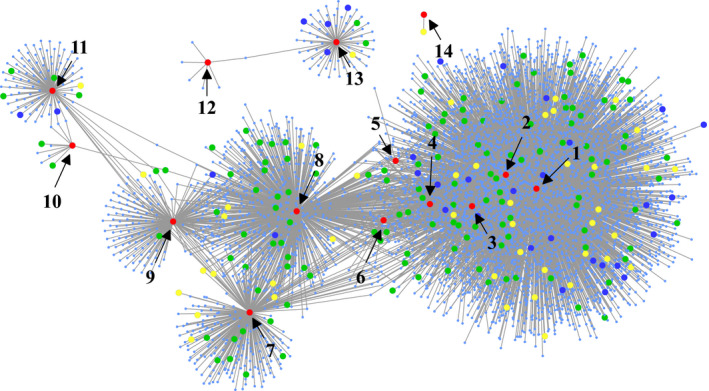
A co‐expression network of the amino acids metabolism. The red nodes represent the 14 candidate genes from GWAS. The yellow nodes represent the co‐expressed genes overlapping with candidate genes of GWAS. The green nodes represent that genes directly or indirectly related to amino acids metabolism. The blue nodes represent the transcription factors. 1, GRMZM2G147191; 2, GRMZM2G009808; 3, GRMZM2G119482; 4, GRMZM2G178826; 5, GRMZM2G010202; 6, GRMZM5G829778; 7, GRMZM2G081886; 8, GRMZM2G090241; 9, GRMZM2G082214; 10, GRMZM2G161868; 11, GRMZM2G169593; 12, GRMZM2G006480; 13, GRMZM2G127308; 14, GRMZM2G036464.

A Pearson correlation was calculated between the expression level of the 23 candidate genes (source genes) and 28 769 genes analysed by RNA‐sequencing from immature kernels (Fu *et al*., 2013). A total of 6641 directed edges connected 14 of the 23 source genes (big red nodes) and were involved in 4670 target genes (*P *≤ 1 × 10^−20^, *r* ≥ 0.5, Figure 4). Among these 4670 genes, 49 genes (including five source genes) were identified by GWAS (big yellow nodes) as well. Another 140 annotated genes (big green nodes), including 33 transcription factors (big blue nodes), were identified to be directly or indirectly associated with amino acid metabolism. GO term analysis of the 4670 co‐expressed genes revealed significant enrichment in terms relating to metabolism, including amine metabolism, cellular processes, developmental processes and biological regulation (Figure S5, Table S5). In addition, we found that four candidate genes (GRMZM2G147191, GRMZM2G009808, GRMZM2G119482, GRMZM2G178826) were related in glycolytic pathway and TCA cycle based their annotation in this co‐expression network (Figure 4).

### Functional validation of candidate genes

**Figure 5:**
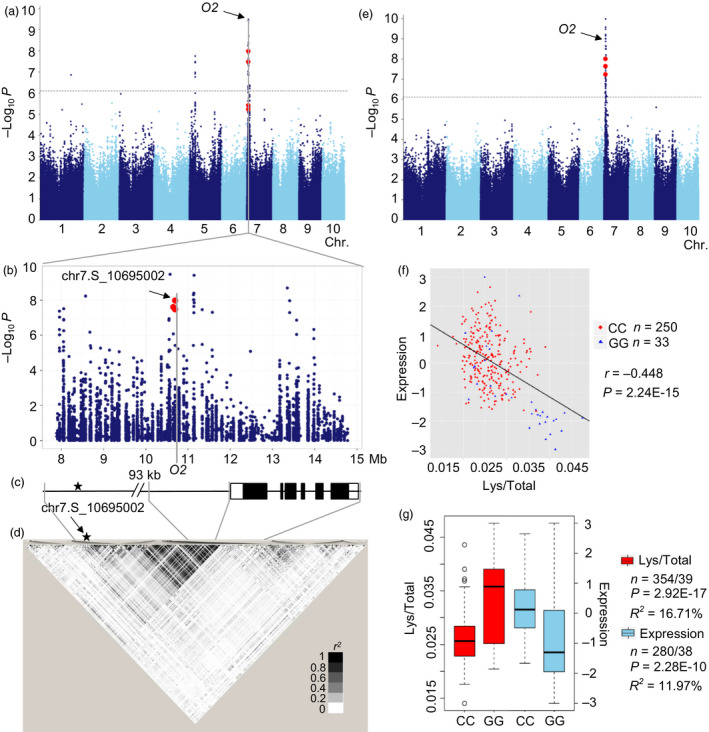
GWAS for Lys/Total with significant SNP‐trait association in this study. (a) Manhattan plot displaying the GWAS result of the Lys/Total level. (b) Regional association plot for locus O2. The SNPs in the promoter and gene body of O2 were shown in red. (c) Gene structure of O2. Filled black boxes represent exons, and filled white ones represent UTRs. (d) A representation of the pairwise *r* ^2^ value among all polymorphic sites in O2, where the colour of each box corresponds to the *r* ^2^ value according to the legend. (e) Manhattan plot shows the association between expression level of O2 and genomewide SNPs. Significant signals are mapped to SNPs within O2, indicating a *cis* transcriptional regulation of this gene. (f) Plot of correlation between the Lys/Total level (red) and the normalized expression level (sky blue) of the O2. The r value is based on a Pearson correlation coefficient. The P value is calculated using the Student's‐*t* test. (g) Box plot for Lys/Total level (red) and expression of O2 (sky blue).

A strong signal (*P* = 1.05 × 10^−8^, *n* = 393) was identified on the short arm of chromosome 7 (Figure 5a), associated with Lys/Total, which could explain 8.5% of the phenotypic variation. The *O2* (GRMZM2G015534) gene is located about 98Kb downstream of the lead SNP chr7.S_10695002 (Figure 5b–d). O2 is a bZIP transcription factor that regulates the expression of various genes during maize kernel development, particularly abundant endosperm storage protein genes like encoding the 22‐kD α‐and β‐zein (Li *et al*., 2015). The lead SNP was strongly associated with the *O2* expression level (*P* = 2.25 × 10^−10^, *R* ^2^ = 11.96%, *n *= 318) and phenotypic trait (*P *= 2.92 × 10^−17^, *R* ^2^ = 16.71%, *n* = 393). Subsequently, a strong *cis*‐eQTL was detected for *O2* (*P *= 1.04 × 10^−10^, MLM, *n *= 368, Figure 5e), and the expression level of *O2* was significantly negatively correlated with the level of Lys/Total ratio (*r *= −0.448, *P *= 2.24 × 10^−15^, *n *= 283, Figure 5f‐g, Table S6). In addition, the significant correlations between the expression levels of *O2* and many other genes were found. The top 2% of genes (575) with the lowest *P*‐value (*P* < 1.0 × 10^−15^) were retained for further analysis including nine genes identified by present GWAS affecting different amino acid traits (Tables S3 and S7). And 22 of 575 genes were also identified by ChIP‐Seq and RNA‐sequencing in *o2* mutant and wide type (Li *et al*., 2015; Table S7). Another 40 genes involved in amino acid metabolism were in the relevant pathways but were not detected by GWAS (Figure S6). These results confirm the importance of *O2* for regulating the amino acid biosynthesis pathway, and the novel candidate genes may help to identify the *o2* modifiers or regulators and to expand the known regulation pathway.

**Figure 6:**
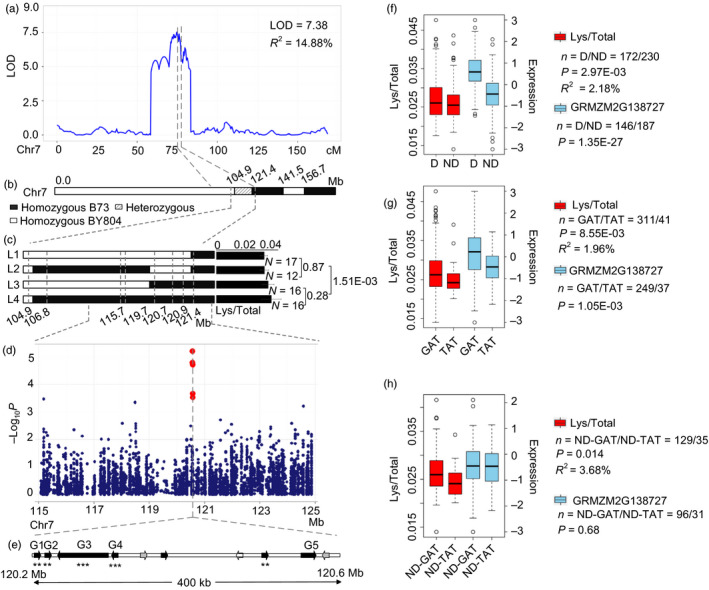
Validation of association analysis using QTL Interval and progeny test. (a) LOD curves of QTL mapping for level of Lys/Total in maize kernels on chromosome 7. (b) Bin map of a heterogeneous inbred family with a heterozygous region on chromosome 7. (c) Progeny test using four progeny families derived from the residual heterozygous line. (d) Scatterplot of association results between SNPs in the confidence interval and the level of Lys/Total. Association analysis was performed using the mixed linear model controlling for the population structure (Q) and kinship (K). (e) The candidate genes of 400 kb in the confidence interval. G1 to G5 represent GRMZM2G138727, GRMZM2G565441, GRMZM2G138976, GRMZM5G873335 and GRMZM2G446625, respectively. *** and *** indicate significant correction between the Lys/Total and the normalized expression levels of candidate genes at *P *<* *0.01 and *P *<* *0.001. (f) Box plot for Lys/Total (red) and expression of GRMZM2G138727 (skyblue) based on duplication (D) and no duplication (ND). (g) Box plot for Lys/Total (red) and expression of GRMZM2G138727 (skyblue) based on B73 (GAT) and By804 (TAT) like haplotype. (h) Box plot for Lys/Total and expression of GRMZM2G138727 based on B73 (GAT) and By804 (TAT) like haplotype within no duplication (ND).

A major QTL on chromosome 7 (LOD = 7.38, *R* ^2^ = 14.88%) affecting Lys/Total was identified in BB RIL population (Figure 6a) with a confidence interval greater than 10 cM (70.4–81.1 cM), and physical length greater than 27 Mb (102.65–129.86 Mb) (Table S4). This QTL was validated in a heterogeneous inbred family (HIF) covering the target region (Figure 6b). Four genotyped and phenotyped progeny families were obtained, which helped to narrow the location of this QTL to a 5.7 Mb region (115.7–121.4 Mb) (Figure 6c). A GWAS signal was detected within the QTL interval located at 120.57 Mb (*P *= 6.26 × 10^−6^, *n *= 393, Figure 6d). Ten candidate genes were obtained within the 400Kb region around the peak including one *zp27* (GRMZM2G138727), two ARID‐transcription factors (GRMZM2G138976 and GRMZM5G873335), one AP2‐EREBP‐transcription factor (GRMZM2G052667) and six unknown genes. GRMZM2G123018 was not detected in RNA‐sequencing of 15 DAP (Fu *et al*., 2013) (Figure 6e, white arrow shown). eQTLs were identified for seven of the nine expressed genes (except GRMZM2G700198 and GRMZM2G003225, Figure 6e). Lys/Total was significantly correlated with the expressions of five of the seven genes (Figure 6e and Table S8) which were then considered as candidate genes. Recently, a QTL (*q*γ*27*) designated *o2 modifier1* in bin 7.02 affecting the expression of 27‐kDa γ‐zein was cloned and co‐localizes with our present locus (Liu *et al*., 2016b). *qγ27* resulted from a 15.26 kb duplication at the 27‐kDa γ‐zein locus contained four genes (GRMZM2G138727, GRMZM2G565441, GRMZM2G138976, and GRMZM5G873335) which overlap with our proposed candidate genes (Figure 6e). We used the primer pair (0707) reported in previous study (Liu *et al*., 2016b) to genotype the association panel and the parents of the BB RIL population. The results showed that this duplication significantly influenced the Lys/Total level (*P *= 2.97 × 10^−3^, *R* ^2^ = 2.18%, *n* = 402) and the expression level of the four candidate genes (Figure 6f, Figure S7, *P* = 1.35 × 10^−27^, *n* = 333). That included this duplication not only influenced the 27‐kDa γ‐zein level, but also influenced the Lys/total level. Surprisingly, a QTL was identified in BB RIL population, but the B73 and By804 did not contain the duplication. This implies that other causal variants may exist within the target gene, in addition to the duplication. Haplotype analysis identified four major haplotypes at GRMZM2G138727 (Figure S8) and a significant difference was observed between B73‐like (GAT) and By804‐like (TAT) haplotypes, both for Lys/Total level (*R* ^2^ = 1.96%, *P *= 8.55 × 10^−3^, *n* = 352) and expression (*P* = 1.05 × 10^−3^, *n *= 286) (Figures 6g, S7). To exclude the possible influence of the duplications, we compared the difference between B73‐like and By804‐like haplotypes within the lines without duplications. Significant association was still observed for Lys/Total level (*R* ^2 ^= 3.68%, *P* = 0.014, *n* = 164) but not for expression (*P* = 0.681, *n* = 127) (Figure 6h), although the sample size was more than halved. Low‐linkage disequilibrium (*r* ^2^ = 0.1) was found between the duplication and the two haplotypes which implies that they were two independent variants and that the gene may affect the phenotype, but not gene expression. Combining effects of the two variants was much greater (*R* ^2^ = 3.74%, *P* = 6.96 × 10^−3^, *n* = 322) than single variant that provided beneficial information for high‐quality maize breeding.

**Figure 7:**
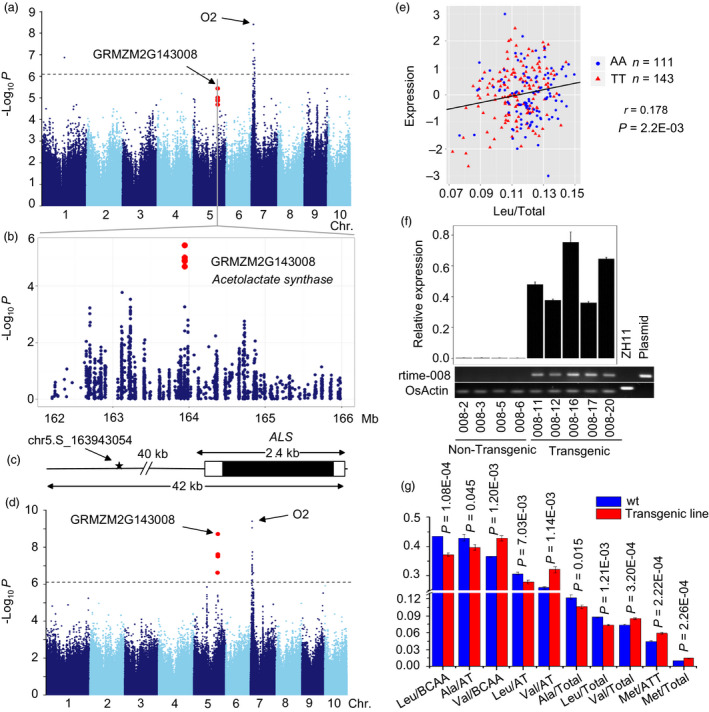
GWAS for Leu/Total with significant SNP‐trait association in this study. (a) Manhattan plot displaying the GWAS result of the Leu/Total level. (b) Regional association plot for locus Acetolactate synthase (GRMZM2G143008). (c) Gene structure of ALS. (d) Manhattan plot shows the association between expression level of GRMZM2G143008 and genomewide SNPs. (e) Plot of correlation between the Leu/Total level (red) and the normalized expression level of the Acetolactate synthase gene (skyblue). The *r* value is based on a Pearson correlation coefficient. The *P* value is calculated using the Student's‐*t* test. (f) The relative expression of GRMZM2G143008 in transgenic and non‐transgenic plants. ZH11 was DNA as the positive control, and plasmid was the over‐expression construct as the negative control. (g) Bar plot for amino acid traits in rice transgenic lines relative to wide type.

*ALS*,* Acetolactate synthase 1* (GRMZM2G143008), located on chromosome 5 and involved in branched‐chain amino acid metabolism, catalyses the first step of Val and Leu biosynthesis. *ALS* was found to associate with Leu/Total (*P *= 3.59 × 10^−6^, *R* ^2 ^= 6.84%, *n *= 394), and the lead SNP (chr5.S_163943054) was located about 41 kb upstream of the *ALS* gene (Figure 7a–c). Two eQTLs including one strong *cis*‐eQTL (*P *= 1.91 × 10^−9^, MLM, *n *= 368, Figure 7d, Tables 2, S3) and one *trans*‐eQTL (*P *=* *3.8 × 10^−10^, MLM, *n *=* *368) were detected for *ALS*. The *trans*‐eQTL was *O2*, which implies that *O2* may regulate the expression of *ALS*. In addition, the aforementioned co‐expression analysis of *O2* and the difference in the expression of genes between *o2* mutant and wide type (Li *et al*., 2015) both identified *ALS* that was regulated by *O2*. *ALS* may affect the trait by regulating the gene expression as the expression level of *ALS* was positively correlated with Leu/Total (*r *=* *0.178, *P *=* *2.20 × 10^−3^, *n *=* *295, Figure 7e) based the phenotype and RNA‐sequencing data of association panel, and this process may be regulated by *O2*, as discussed above. Consequently, we overexpressed *ALS* in rice and a significant difference was observed between the transgenic (Figure 7f) and nontransgenic plants for a number of traits including Leu/Total, Val/BCAA, Val/Total, Val/TA and others involved in the branched‐chain amino acids pathway (Figure 7g). The nontransgenic plants had higher Leu/BCAA, Leu/AT and Leu/Total level than the transgenic ones, but the Val/BCAA, Val/Total and Val/TA involved in the same metabolic pathway increased in transgenic plants. According to the previous study (Binder, 2010), the *ALS* catalyses the first step in the parallel pathway towards Val/Leu and Ile in Arabidopsis. Here, we observed a significant difference in Val, Leu, Ala, and Met between transgenic and nontransgenic lines, but not in Ile. More studies are still required to fully explore the biosynthesis of branched‐chain amino acids.

## Discussion

Amino acids provide essential building blocks for proteins and act as signalling molecules during plant germination, growth, development and reproduction. Grain proteins are the major source of essential amino acids in food and feed. Amino acid biosynthesis is not fully elucidated in higher plants as compared to bacteria (Umbarger, 1969, 1978) and most of the information has been from model plant Arabidopsis (Coruzzi, 2003; Hell and Wirtz, 2011; Ingle, 2011; Jander and Joshi, 2009; Tzin and Galili, 2010a,b; Verslues and Sharma, 2010). In this study, GWAS and linkage mapping were used to dissect the genetic basis of amino acid content in mature maize kernel. We identified 528 loci and 404 QTLs through GWAS and linkage mapping, respectively. Most of the identified loci or QTLs had moderate effects, explaining between 5% and 15% of the phenotypic variation (Figure S3, Tables S3, S4). Similar results have also been reported in other metabolite studies in maize (Riedelsheimer *et al*., 2012; Wen *et al*., 2014, 2015, 2016). It is only a few QTLs (15/404) could be identified in multiple RIL populations, implying that QTLs affecting amino acid composition were genetic background dependent. On average, 5.1 and 4.4 loci per trait were identified using GWAS in AM1 and AM2, respectively, and some of them were located within the identified QTLs (17/528). It appears that the genetic basis of amino acid content in the maize kernel is relatively be simple and controlled by few genes compared with other complex quantitative traits, including agronomic traits (Xiao *et al*., 2016). A co‐expression network was constructed based on the genes identified by GWAS and gene expression data in kernel of 15 DAP (Figure 4) and novel genes involved were found. These genes are enriched in different metabolic processes and may function as downstream and/or upstream regulators. Further studies are required to fully explore the genetic control of amino acid biosynthetic pathways.

QTLs were not randomly distributed on the chromosomes, with eight QTL hotspots observed (Figure 2) on four different chromosomes. The underlying genes were not identified for most of the QTL hotspots. This kind of QTL clustering was also observed in other maize studies (Riedelsheimer *et al*., 2012; Wen *et al*., 2015; Zhang *et al*., 2015) and in other plants: tomato (Causse *et al*., 2002; Schauer *et al*., 2008), rice (Chen *et al*., 2014; Gong *et al*., 2013; Matsuda *et al*., 2012) and *Arabidopsis* (Lisec *et al*., 2008). This could be explained by the joint effects of closely linked genes (in local LD) (Bergelson and Roux, 2010) or by pleiotropy. Two QTL hotspots that affect many different phenotypic traits was identified on chromosome 7 (Figure 2). *O2* is located in one of the two QTL hotspots and appears to regulate many other genes, as identified by co‐expression analysis (Figure S6). In a recent study, up to 35 *O2*‐modulated target genes were identified by RNA‐sequencing and ChIP‐sequencing based on the *o2* mutant (Li *et al*., 2015), some of which overlapped with our findings (Figure S6, Table S7). *o2* mutants have higher lysine content but usually worse agronomic performance, limiting their commercial utility. The materials used in the present study are all elite inbred lines with normal field performance, differing in amino acid content, including lysine, implying that natural genetic variation in *O2* and other genes existing in the maize germplasm could be used for the improvement of amino acid composition in the future. Identification of the favourable alleles affecting amino acid composition for enhancing high nutritional maize breeding is an important priority.

The quality protein maize (QPM) was developed by introducing the *o2 modifier(s)* into *o2* maize (Lopes *et al*., 1995) and has normal phenotype and yield, but the high lysine content of the *o2* mutant. However, the breeding process is time‐consuming, and the mechanism and genetic architecture of *o2 modifiers* is poorly understood. Seven *o2 modifiers* have been located using a F_2_ population (Holding *et al*., 2008). More recently, one of the modifiers, *qγ27,* was cloned and gene duplication was found to increase the expression of 27‐kDa γ‐zein, affecting protein content (Liu *et al*., 2016b). It was confirmed that this duplication is also present in our diverse maize inbred collections and affects the Lys/Total level and lysine content. It is interesting that a QTL was also identified in the BB RIL populations, whose parents did not contain this duplication. Additional causal variation exists within *q*γ*27* and was not in linkage disequilibrium with the duplication may provide new alleles for future quality protein maize breeding.

## Materials and methods

### The association panel and RIL populations

Genetic materials used in this study included an association panel of 513 diverse maize inbred lines for GWAS (Li *et al*., 2012b; Yang *et al*., 2011, 2014) and three recombinant inbred line (RIL) populations B73/BY804 (BB), ZONG3/YU87‐1 (ZY) and KUI3/B77 (KB) for linkage analysis (Pan *et al*., 2016; Xiao *et al*., 2016). The association panel was composed of tropical, subtropical and temperate materials representing global maize diversity; details were described in previous studies (Li *et al*., 2012b; Yang *et al*., 2011, 2014). Field trials for the association panel were conducted in two environments: Yunnan (N 24 25′, E 102 30′) in 2011 and Chongqing (N 29 25′, E 106 50′) in 2012. RIL populations were phenotyped in three environments. The 197 RILs from BB were planted in Hainan (N 18 25′, E 109 51′) in 2011, and the 197 RILs from ZY and 177 RILs from KB were planted in Yunnan (N 24 25′, E 102 30′) in 2011. An incompletely randomized block design was used for the field trials of all the inbred lines including the association panel and three RIL populations, and a single replicate was conducted in each location. All lines were self‐pollinated and five ears were harvested from each plot at maturity and were air‐dried and shelled. A mixture of kernels from five self‐pollinated ears was used to measure the amino acids.

### Genotypes

The association panel was genotyped using Illumina MaizeSNP50 BeadChip (Ganal *et al*., 2011) and a genotyping by sequencing method (Elshire *et al*., 2011). Kernels from five immature ears of 368 maize inbred lines were collected at 15 days after self‐pollination for RNA extraction. 1.03 million high‐quality SNPs and the expression data of 28 769 genes were obtained by RNA‐sequencing, (Fu *et al*., 2013; Li *et al*., 2013). Affymetrix Axiom Maize 600K array (Unterseer *et al*., 2014) was used to genotype 153 lines of the association panel. After strict quality controls for each dataset, the genotypes from four different genotyping platforms were merged and 1.25M SNPs with a MAF> = 5% were used for further studies (Liu *et al*., 2016a). The three RIL populations were also genotyped by Illumina MaizeSNP50 BeadChip and high‐density linkage maps were constructed with 2496, 3071, and 2126 unique bins for BB, ZY and KB, respectively (Pan *et al*., 2016; Xiao *et al*., 2016).

### Amino acids analysis

The amino acid concentrations of the matured maize kernel from the association panel and the three RIL populations were determined using an automatic amino acid analyzer L‐8800 (L‐8800, Hitachi Instruments Engineering, Tokyo, Japan). About 50–70 mg per sample of seed powder was used for the total amino acids analysis. Each sample was solubilized in 10 mL 6 M HCl at 110° for 22 h. To remove the insoluble materials, all samples were filtered into a 50‐mL volumetric flask, then deionized water was added to 50 mL and mixed well. 750 μL mix of each sample was transferred to a 2‐mL tube and evaporated. The dried materials were then re‐dissolved in 750 μL 0.02N HCl. Subsequently, 20 μL of the re‐dissolved materials were injected into an automatic amino acid analyser and the raw data was analysed with L‐8800 software ASM (Zhou *et al*., 2009). Finally, the levels of seventeen amino acids of mature maize kernel (Ala = Alanine, Arg = Arginine, Asx = Aspartic acid and Asparagine, Glx = Glutamine and Glutamic acid, Gly = Glycine, Ile = Isoleucine, Leu = Leucine, Lys = Lysine, Met = Methionine, Pro = Proline, Phe = Phenyalanine, Val = Valine, Tyr = Tyrosine, His = Histidine, Cys = Cysteine, Thr = Threonine and Ser = Serine in mg/g dry maize kernel) and the total amino acid content (sum of the seventeen amino acids) were obtained using this method. Forty‐seven derived traits were determined: aspartate‐derived amino acid (abbreviated ATT, included Lys, Asx, Met, Ile and Thr), pyruvate‐derived amino acid (abbreviated AT, included Ala, Leu and Val), the branched‐chain amino acid (abbreviated BCAA, included Ile, Leu and Val), serine‐derived amino acid (abbreviated ST, Ser, Gly and Cys), phenylalanine‐derived amino acid (abbreviated PT, Phe and Tyr), glutamate‐derived amino acid (abbreviated GT, included Glx, Pro and Arg) (Table S1). Each amino acid content was expressed as a percentage of the total amino acid, and the ratio of each relative amino acid content to the sum of corresponding derived amino acids were the derived traits, including Ala/Total, Arg/Total, Asx/Total, Glx/Total, Gly/Total, Ile/Total, Leu/Total, Lys/Total, Met/Total, Pro/Total, Phe/Total, Vla/Total, Tyr/Total, His/Total, Cys/Total, Thr/Total, Ser/Total, Lys/ATT, Asx/ATT, Met/ATT, Ile/ATT, Thr/ATT, Ala/AT, Leu/AT, Val/AT, Ile/BCAA, Leu/BCAA, Val/BCAA, Ser/ST, Gly/ST, Cys/ST, Phe/PT, Tyr/PT, Glx/GT, Pro/GT and Arg/GT.

### Genomewide association study

A genome wide association study (GWAS) was conducted for maize kernel amino acid traits. To test the statistical associations between genotype and phenotype, a mixed linear model was used for accounting for the population structure and relative kinship (Li *et al*., 2013; Yu *et al*., 2006). Considering the maker number in present study is 1.25 million and many of them should be in linkage disequilibrium. The effective number of independent marker (*N*) was calculated using the GEC software tool (Li *et al*., 2012a). Suggestive (1/*N*) *P* value thresholds were set to control the genomewide type 1 error rate. The suggestive value was 2.04E‐06 for whole population and used as the cut‐offs. The *P* value of each SNP was calculated using Tassel3.0. For all traits, the lead SNP (SNP with the lowest p value) at an associated locus and its corresponding candidate genes in or near (within 100 kb up‐, downstream of the lead SNP) known genes were reported (Table S3). If the associated SNPs were not in or near an annotated amino acid metabolism gene, the closest of the lead SNP candidate gene was considered the most likely candidate gene. The physical locations of the SNPs were based on the B73 RefGen_v2.

### QTL mapping

The linkage mapping was conducted using Composite Interval Mapping (CIM) implemented in Windows QTL Cartographer V2.5 (Wang *et al*., 2006; Zeng *et al*., 1999) for all amino acid traits measured in maize kernel of the three RIL populations. The methods followed the Windows QTL Cartographer V2.5 user manual. Zmap (model 6) with a 10‐cM window and a walking speed of 0.5 cM was used. For each trait, a uniform threshold for significant QTLs was determined by 500 permutations (*P* = 0.05). The parameter was set as default. 2.0 LOD–drop confidence interval was used for each QTL as described.

In total, 13 progeny families were derived from one heterogeneous inbred line that were identified for the major QTL on chromosome 7 and planted at Wuhan in the summer of 2014 for QTL validation and cloning. Six families with enough seeds (*n* = 10 to 25 rows, 11 individuals per row for each family) were planted at Hainan in the winter of 2014. Two families (*n* = 29 and 32 individuals for each family) with enough recombinant individuals were measured for amino acids with one replicate. Primers used for linkage analysis were listed in Table S9.

### eQTL mapping

Expression mapping (eQTL) analysis (SNP vs. gene expression level) used the same method described above for GWAS. The association analysis between the genomewide SNPs and the identified candidate gene expression level was performed. Only those genes expressed in more than 50% of 368 lines and for which at least 10 reads were available were used in this analysis (Liu *et al*., 2016a).

### Co‐expression network

In order to construct the co‐expression network of chosen genes, we calculated pairwise relative expression coefficients in R (https://www.r-project.org/) and used these coefficients and *P*‐values to filter the genes. The filtered co‐expression genes were used to construct the co‐expression network. The pairwise relative expression coefficients shown the relationship between genes. The program Cytoscape was used to draw the network with only the most highly connected genes (http://www.cytoscape.org/). The Gene Ontology term analysis was conducted at AGRiGO (http://bioinfo.cau.edu.cn/agriGO/).

### Plasmid construction and rice transformation

The overexpression vector pCAMBIA1300nu was provided by Dr. Yongjun Lin, Huazhong Agricultural University, Wuhan, China. To generate the GRMZM2G143008 over‐expression construct, the open reading frame of GRMZM2G143008 was amplified from the cDNA of maize inbred line B73 developing kernel by PCR using the gene‐specific primers DMp008Os‐F and DMp008Os‐R, which contained a 20‐bp fragment complementary with pCAMBIA1300nu. The PCR product was cloned into pCAMBIA1300nu with a homologous recombination clone kit (Vazyme, China). The target gene was driven by a maize ubiquitin promoter. Then the correct clone was selected by sequencing the construct. These constructs were introduced into *japonica* rice cultivar ZhongHua 11 (ZH11) by *Agrobacterium tumefaciens*‐mediated transformation (Lin and Zhang, 2005). Primers used in this study were listed in Table S9.

### Expression analysis of transgenic plant

Total RNA was prepared from leaves using a Quick RNA Isolation kit (HUAYUEYANG, Beijing). For RT‐PCR, the first‐strand cDNA was synthesized from 1.5 mg total RNA using the TransScript One‐Step gDNA Removal and cDNA Synthesis SuperMix kit (TransGen, China). Semi‐quantitative PCR was performed for gene expression analysis using gene‐specific (DMp008Os‐F and DMp008Os‐R) and rice ACTIN (OsrActin‐F and OsrActin‐R) primers. Real‐time PCR was performed on an optical 96‐well plate in a BIO‐RAD CFX96 Real‐Time system using TransStart Tip Green qPCR SuperMix (TransGen, China). Actin was used as an endogenous control. Primers used in this study were listed in Table S9.

## Supporting information


**Figure S1** Fold difference of amino acids levels within AM1 and AM2 association panels, and the B73/By804 (BB), Kui3/B77 (KB) and Zong3/Yu87‐1 (ZY) RIL populations.
**Figure S2** The QTL/loci number distribution per trait in 2011 Yunnan (AM1) and 2012 Chongqing (AM2) association panels, and the B73/By804 (BB), Kui3/B77 (KB) and Zong3/Yu87‐1 (ZY) RIL populations, respectively.
**Figure S3** Phenotypic variation explained for each identified locus or QTL in 2011 Yunnan (AM1) and 2012 Chongqing (AM2) association panels, and the B73/By804 (BB), Kui3/B77 (KB) and Zong3/Yu87‐1 (ZY) RIL populations, respectively.
**Figure S4** Gene Ontology term analysis of GWAS candidate genes.
**Figure S5** Gene Ontology annotation of 4670 co‐expression genes from 14 GWAS candidate genes.
**Figure S6** The Opaque2 regulated network.
**Figure S7** Box plot for the expression level of GRMZM2G138727, GRMZM2G565441, GRMZM2G138976 and GRMZM5G873335 based on duplication (D), no duplication (ND), B73‐like (GAT), and By804‐like (TAT) haplotypes.
**Figure S8** Gene structure and LD block of GRMZM2G138727.

**Table S1** Statistical summary of 65 amino acid traits in maize kernels in the association panel.
**Table S2** Statistical summary of 65 amino acid traits in maize kernels in RIL populations.
**Table S3** Significant loci associated with amino acid traits identified by GWAS across two environments.
**Table S4** QTL mapping summary of amino acid‐related traits detected from three RIL populations.
**Table S5** The list of significant Gene Ontology terms.
**Table S6** The Lys/Total, Lys content and *O2* expression level in the association mapping panel.
**Table S7** *O2* co‐expression genes overlapped with ones identified by other methods.
**Table S8** The eQTL of ten candidate genes and the Pearson correction between Lys/Total and the normalized expression levels of ten candidate genes.
**Table S9** Primers used for mapping, plasmid construction, and expression analysis.
