# Supplementary material for: The genetic architecture of amino acids dissection by association and linkage analysis in maize
Source: Plant Biotechnol J. 2017 Apr 5;15(10):1250–63. doi: 10.1111/pbi.12712 (PMC5595712; doi:10.1111/pbi.12712)

**Supporting Information Figures**

**Supplementary Figure1.** Fold difference of amino acids levels distribution within AM1 and AM2 association panels, and the B73/By804 (BB), Kui3/B77 (KB) and Zong3/Yu87-1 (ZY) RIL populations, respectively.


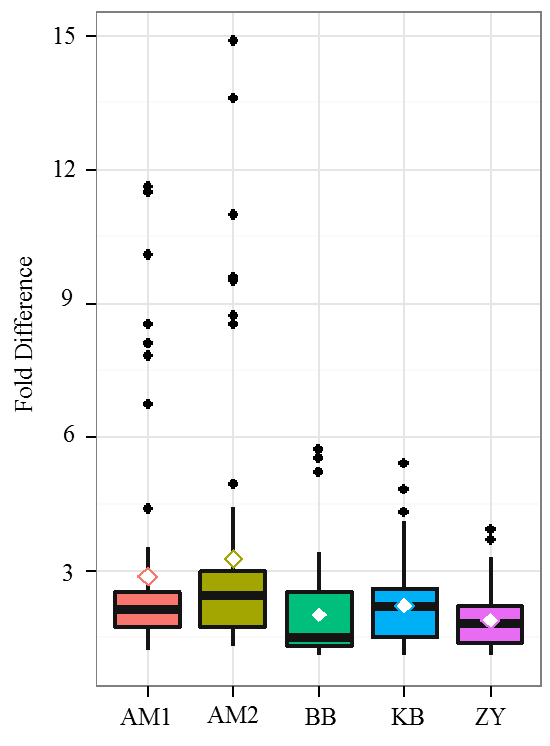


**Supplementary Figure 2.** The QTL/loci number distribution per trait in 2011 Yunnan (AM1) and 2012 Chongqing (AM2) association panels, and the B73/By804 (BB), Kui3/B77 (KB) and Zong3/Yu87-1 (ZY) RIL populations, respectively.

**Supplementary Figure 3.** The phenotypic variation explained distribution of each identified locus or QTL in 2011 Yunnan (AM1) and 2012 Chongqing (AM2) association panels, and the B73/By804 (BB), Kui3/B77 (KB) and Zong3/Yu87-1 (ZY) RIL populations, respectively.


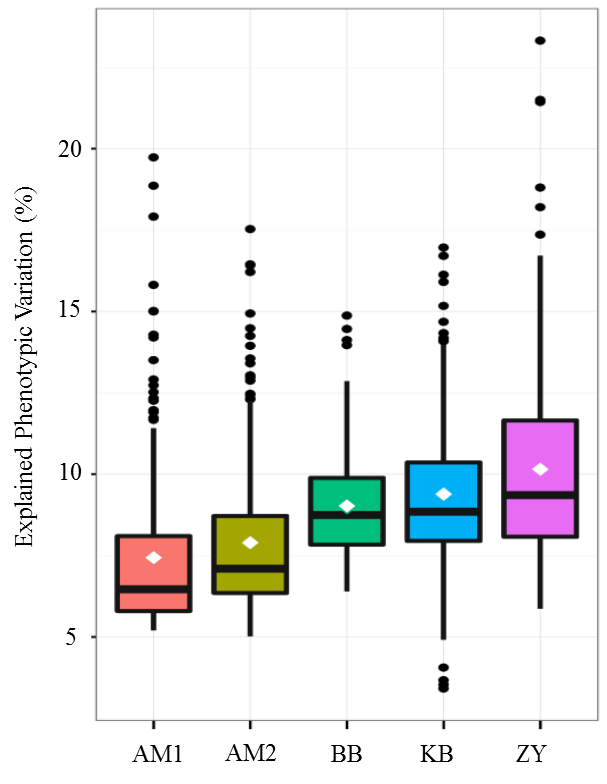


**Supplementary Figure 4.** The Gene Ontology term analysis of GWAS candidate genes.


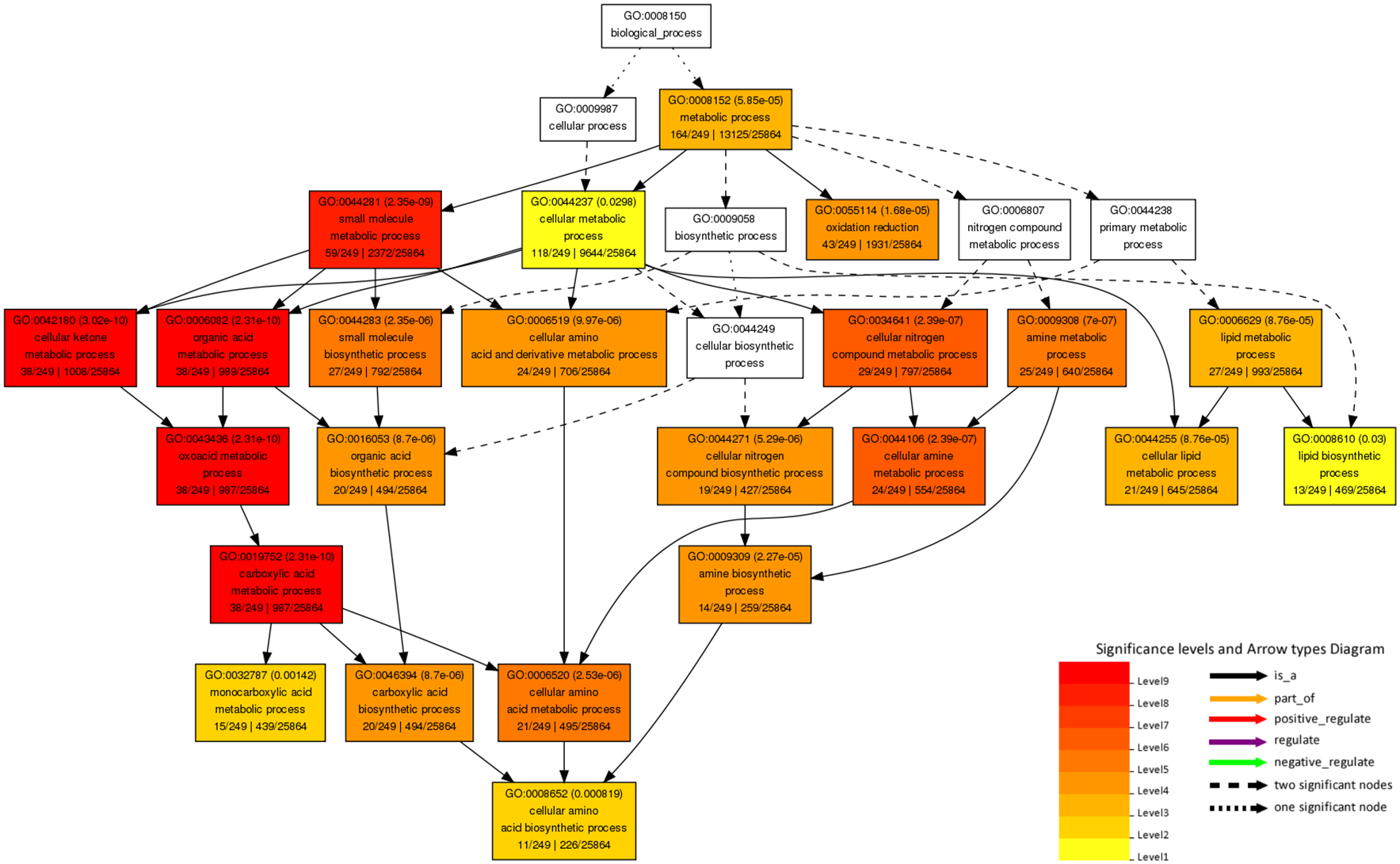


**Supplementary Figure 5.** The Gene Ontology annotation of 4,670 co-expression genes from 14 GWAS candidate genes.


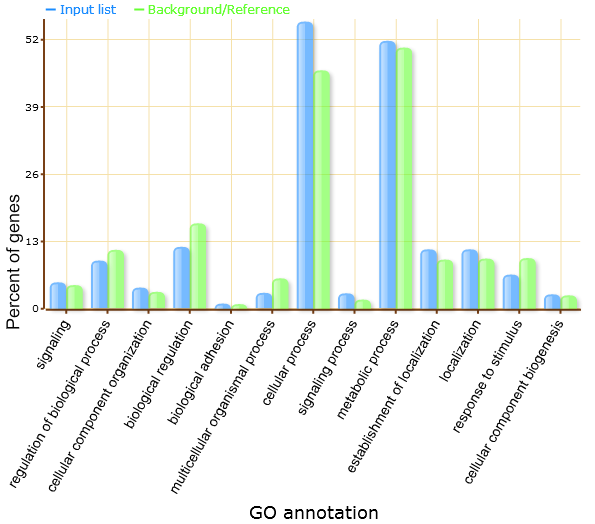


**Supplementary Figure 6.** The Opaque2 regulated network. (a) The network of the top 2% genes of the lowest p-value. The green dots mean the identified genes in present or previous studies. The yellow dots mean the identified genes related amino acids metabolism. The pink dots mean the other genes. (b) The network of identified genes related amino acids metabolism. The green dots mean the identified genes in present or previous studies. The yellow dots mean the identified genes related amino acids metabolism.


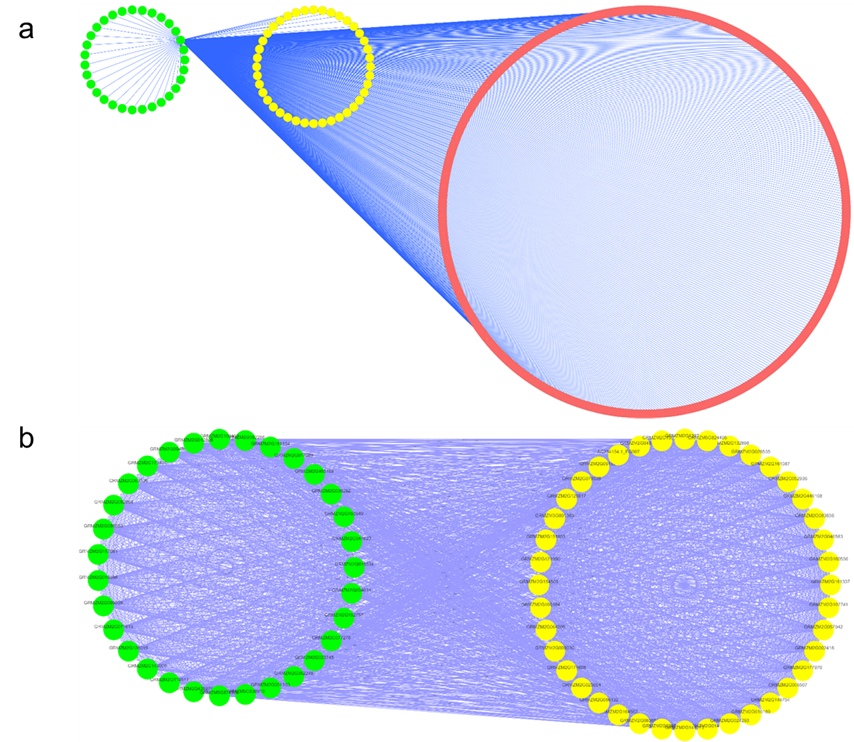


**Supplementary Figure 7.** Box plot for the expression level of GRMZM2G138727, GRMZM2G565441, GRMZM2G138976 and GRMZM5G873335 based on duplication (D), no duplication (ND), B73-like (GAT) and By804-like (TAT) haplotypes.

**Supplementary Figure 8.** Gene structure and LD block of GRMZM2G138727. (a) The gene structure of GRMZM2G138727. (b) A representation of the pair-wise r2 value among all polymorphic sites in GRMZM2G138727. (c) The haplotype analysis.


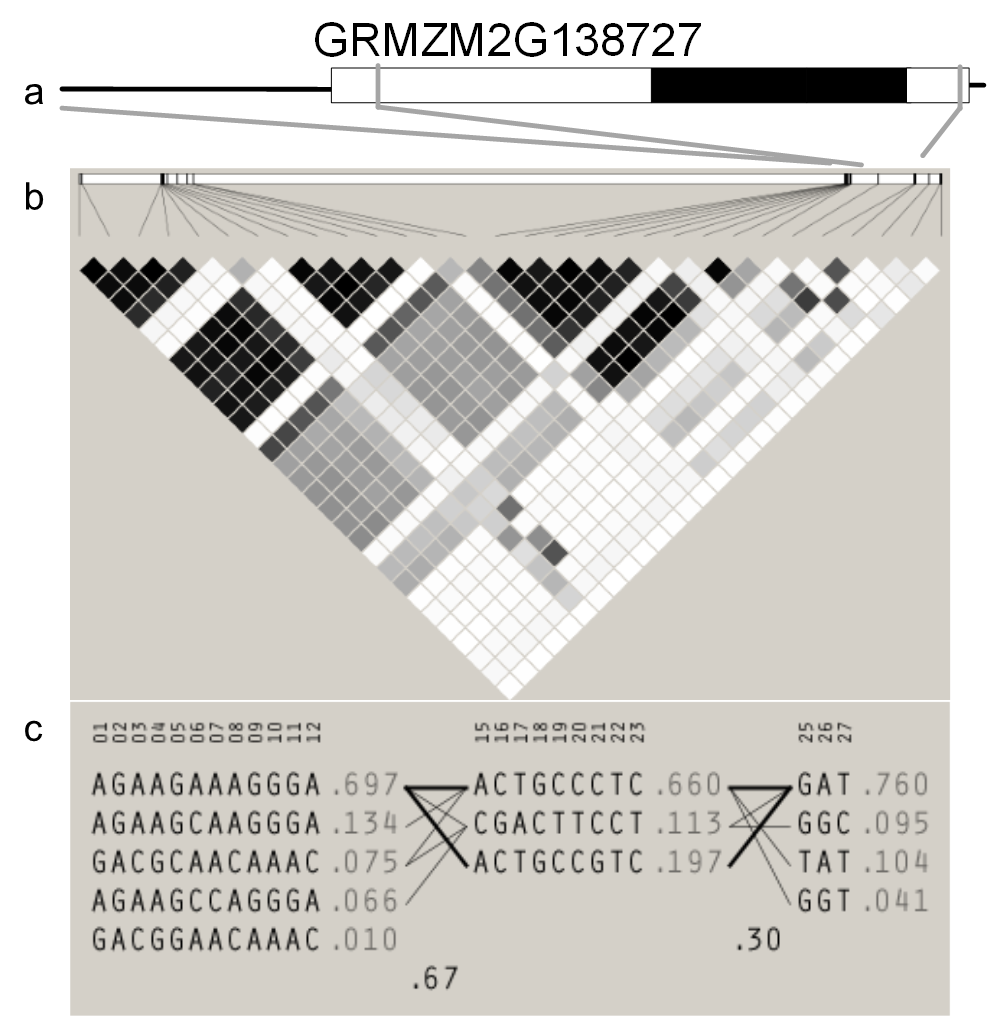

Supplement: Supplementary file 1 — Figure S1 Fold difference of amino acids levels within AM1 and AM2 association panels, and the B73/By804 (BB), Kui3/B77 (KB) and Zong3/Yu87‐1 (ZY) RIL populations. Figure S2 The QTL/loci number distribution per trait in 2011 Yunnan (AM1) and 2012 Chongqing (AM2) association panels, and the B73/By804 (BB), Kui3/B77 (KB) and Zong3/Yu87‐1 (ZY) RIL populations, respectively. Figure S3 Phenotypic variation explained for each identified locus or QTL in 2011 Yunnan (AM1) and 2012 Chongqing (AM2) association panels, and the B73/By804 (BB), Kui3/B77 (KB) and Zong3/Yu87‐1 (ZY) RIL populations, respectively. Figure S4 Gene Ontology term analysis of GWAS candidate genes. Figure S5 Gene Ontology annotation of 4670 co‐expression genes from 14 GWAS candidate genes. Figure S6 The Opaque2 regulated network. Figure S7 Box plot for the expression level of GRMZM2G138727, GRMZM2G565441, GRMZM2G138976 and GRMZM5G873335 based on duplication (D), no duplication (ND), B73‐like (GAT), and By804‐like (TAT) haplotypes. Figure S8 Gene structure and LD block of GRMZM2G138727. [file PBI-15-1250-s002.docx]
